# *N*-benzyl-*N*-methyldithiocarbamate (BMDC) combines with metals to produce antimicrobial and anti-biofilm activity against methicillin-resistant *Staphylococcus aureus* (MRSA) and *Staphylococcus epidermidis*

**DOI:** 10.1128/msphere.00691-25

**Published:** 2025-12-11

**Authors:** Yamil Sanchez-Rosario, Natasha R. Cornejo, Isaiah S. Gonzalez, Vanessa Brizuela, Klariza Ochoa, Chloe Scott, Michael D. L. Johnson

**Affiliations:** 1 Department of Immunobiology, University of Arizona College of Medicine - Tucson 242724, Tucson, Arizona, USA; 2 Valley Fever Center for Excellence, University of Arizona College of Medicine - Tucson 733661, Tucson, Arizona, USA; 3 BIO5 Institute, University of Arizona College of Medicine - Tucson 124486, Tucson, Arizona, USA; 4 Asthma and Airway Disease Research Center, University of Arizona College of Medicine - Tucson 577409, Tucson, Arizona, USA; University of Nebraska Medical Center College of Medicine, Omaha, Nebraska, USA

**Keywords:** biofilm, copper, zinc, dithiocarbamate, MRSA, *Staphylococcus epidermidis*

## Abstract

Methicillin-resistant *Staphylococcus aureus* (MRSA) is a high-priority microorganism that necessitates the development of new treatments, as it causes a substantial disease burden and economic impact globally. MRSA colonizes the skin and anterior nares and can potentially become invasive, leading to pneumonia and soft tissue infection. Additionally, MRSA can establish chronic infections in wounds and medical implants, partly due to its ability to form biofilms. Likewise, the skin commensal *Staphylococcus epidermidis* also causes similar infections, particularly through its ability to form a plastic-binding biofilm. In this study, we used *N*-benzyl-*N*-methyldithiocarbamate (BMDC) in combination with copper or zinc to decrease the viability of MRSA in both planktonic and biofilm settings *in vitro*, as well as to inhibit biofilm formation by *S. epidermidis*. We used Inductively Coupled Plasma Optical Emission Spectrometry (ICP-OES), biomass assessment, colony counts, and metabolism assays to interrogate the state of the bacterium after exposure to metal-BMDC. Furthermore, we compared these metal complexes to the antibiotic vancomycin, one of the current therapeutics used to treat MRSA infections. BMDC enhances copper uptake in bacteria, increasing intracellular copper levels by 70-fold compared to copper alone. Copper intoxication leads to a decrease in metabolic activity that ultimately results in bacterial death. Zinc also combines with BMDC, though likely through a different mechanism, and similarly exerts bactericidal effects. Significantly, both metal-BMDC combinations effectively reduce biofilm formation and eradicate bacteria within established biofilms *in vitro*, highlighting their potential as promising antimicrobial strategies against MRSA and *S. epidermidis* biofilms.

**IMPORTANCE:**

Antimicrobial-resistant bacteria, such as *Staphylococcus aureus* (MRSA) and *Staphylococcus epidermidis*, are a significant cause of morbidity and mortality in vulnerable populations, contributing to an escalating health and economic burden. Biofilms are an important reservoir that protects bacteria from immune clearance and antimicrobial agents. However, current strategies to effectively target MRSA biofilms are limited. This research describes a therapeutic approach that can disrupt biofilms in both MRSA and *S. epidermidis*, thereby enhancing bacterial clearance.

## INTRODUCTION

*Staphylococcus aureus* is a Gram-positive opportunistic pathogen that colonizes the nose and skin of 20%–30% of the population (1, 2). Colonization can lead to diseases such as soft tissue infections, bacteremia, and pneumonia, which have high morbidity and mortality rates in some populations (3–6). Furthermore, *S. aureus* can infect medical implants by forming biofilms (7, 8). Biofilms are three-dimensional structures composed of extracellular DNA, polysaccharides, and proteins that protect bacteria from antibiotics, the immune system, and environmental stress. Studies have shown that biofilm-associated bacteria are more resistant to environmental stressors (e.g., desiccation and pH changes) and antimicrobial treatments, making eradication of infections challenging (9–11). Moreover, the ability of Methicillin-resistant *Staphylococcus aureus* (MRSA) to form biofilm enables chronic recalcitrant colonization of medical implants and bone (12), exacerbating clinical outcomes. There are multiple strategies to combat biofilms, including phage therapy, antimicrobial-loaded nanoparticles, and various metabolic inhibitors, as well as drug repurposing (13–17). However, because Staphylococcus infections can occur in multiple niches, each approach to combat biofilms faces challenges, and no single method has proven effective in eradicating biofilm-associated MRSA infections (18).

While MRSA commands significant attention, the skin and mucous membrane commensal *Staphylococcus epidermidis* is implicated in infections, particularly in immunocompromised individuals and in association with medical implants (19). Many joint replacements for degenerative joint diseases are coated with ultra-high molecular weight polyethylene due to the material’s durability, hydrophobicity, and low friction. However, despite these qualities, joint replacements remain susceptible to bacterial colonization, especially *S. epidermidis,* due to its exceptional ability to adhere to plastic and medical-grade polymers (20, 21). Such infections can lead to implant failure, resulting in increased patient morbidity and healthcare costs. Furthermore, like MRSA, it forms biofilms through similar mechanisms (22). Additionally, linezolid-resistant varieties have emerged, as reported by the CDC (23–25).

Metals such as iron, calcium, manganese, and zinc are essential cofactors for enzymes and, therefore, necessary for microbial growth within the host. As such, during infection, in a process termed nutritional immunity, the host restricts these metals from bacteria via host protein complexes like calprotectin and lactoferrin (26–28). The acquisition of essential metal nutrients is crucial for bacterial survival, as evidenced by the sophisticated systems bacteria use to scavenge these nutrients from their environment. However, while these metals are indispensable for cellular function, their intracellular accumulation must be tightly regulated. According to the Irving-Williams series, certain metals bind more strongly to biological ligands, and when present in excess, they can disrupt cellular processes, leading to metal intoxication. This underscores the delicate balance in metal homeostasis, where the threshold between sufficiency and toxicity is remarkably narrow. The detrimental effects of metal overload become evident when genes for metal exporters are deleted, resulting in heightened sensitivity to metal exposure (29).

Nevertheless, metal exporters exist to maintain metal homeostasis and are unlikely to be lost in real-world conditions. Therefore, to induce metal intoxication in pathogens, the host must circumvent bacterial metal resistance mechanisms. For instance, *S. aureus* produces the metallophore staphylopine, which can chelate a range of first-row transition metals but is deployed under zinc-limiting conditions as a zinc scavenger (30). However, this zinc scavenger system is also vulnerable to copper binding (31). In response, the bacteria activate the copper export system to maintain bacterial metal homeostasis. This example illustrates how manipulating the host’s metallo-environment potentiates metal dysregulation in bacteria, promoting metal intoxication.

The immune response mobilizes copper and zinc to sites of infection through macrophages and neutrophils to fight off microorganisms (32–34). Consequently, these metals show promise as antimicrobial agents (35, 36), as their excess disrupts multiple cellular pathways simultaneously. We have previously reported that *N*,*N*-dimethyl dithiocarbamate (DMDC) enhances copper toxicity by increasing intracellular copper levels, resulting in effective antimicrobial activity both *in vitro* and *in vivo* (37). Furthermore, we demonstrated that DMDC derivatives increased bacterial intracellular copper levels, resulting in copper intoxication in *Streptococcus pneumoniae* (38). In the current study, we introduce a strategy against MRSA and *S. epidermidis* biofilms by utilizing *N*-benzyl-*N*-methyldithiocarbamate (BMDC) (Fig. S1), a derivative of DMDC, thereby targeting both planktonic and biofilm populations.

## RESULTS

### Metal-BMDC-mediated antimicrobial effects against MRSA

**Fig 1:**
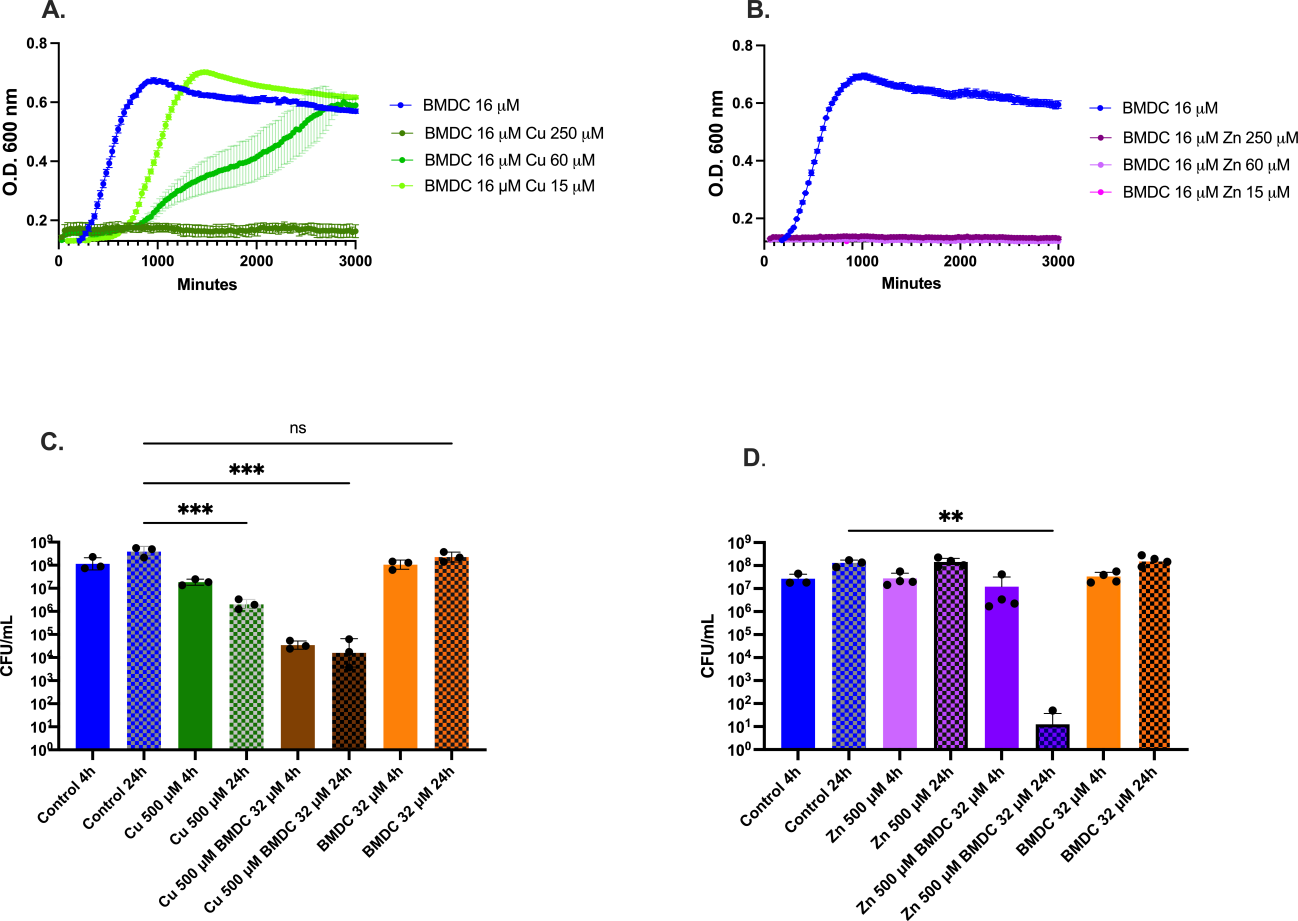
Effect of BMDC combined with copper or zinc on MRSA growth and survival. BMDC combines with metals to enhance antimicrobial efficacy. (**A**) Growth curve of MRSA comparing growth rate under copper excess with 16 µM BMDC. (**B**) Growth curve of MRSA in supplemented RPMI with 16 µM BMDC and increasing concentrations of zinc. (**C**) Survival assay of MRSA in RPMI under metal treatments. MRSA was cultured to a cell density of 10^8 CFU/mL, followed by exposure to copper, BMDC, or a combination of both. At various time points, samples were collected and plated on blood agar plates to assess bacterial survival. (**D**) Same as (**C**) but with zinc instead of copper. Statistical differences were measured using a one-way ANOVA with Tukey’s multiple comparisons test (ns, non-significant; *, *P* < 0.05; **, *P* < 0.01; ***, *P* < 0.001; ****, *P* < 0.0001).

After evaluating the bactericidal effects of multiple concentrations of copper or zinc against MRSA proliferation and finding them to be either ineffective or modestly effective in delaying MRSA growth (Fig. S2A and B), we tested them in combination with BMDC. Bacteria exposed to 16 µM BMDC and various concentrations of copper exhibited a metal-dependent growth inhibition as compared to no treatment. In contrast, the zinc-BMDC treatment resulted in complete growth inhibition to a 1:0.94 ratio of compound to metal (Fig. 1A and B).

Next, we evaluated the bactericidal effects of copper or zinc in combination with BMDC on actively growing MRSA. Copper-BMDC treatment reduced bacterial counts after 4 h of exposure, with bacteriostatic activity persisting for 24 h (Fig. 1C). In contrast to the copper-BMDC, the zinc-BMDC treatment showed little effect at 4 h; however, 24 h later, bacterial counts were below the detection limit. We found one colony on one plate, while seven other plates failed to produce growth in four independent experiments (Fig. 1D). Further experiments demonstrated that the zinc-BMDC treatment led to a gradual bactericidal effect and combining it with copper after 4 h both enhanced and expedited bactericidal activity (Fig. S2C).

### Mechanisms of metal-compound-induced toxicity

**Fig 2:**
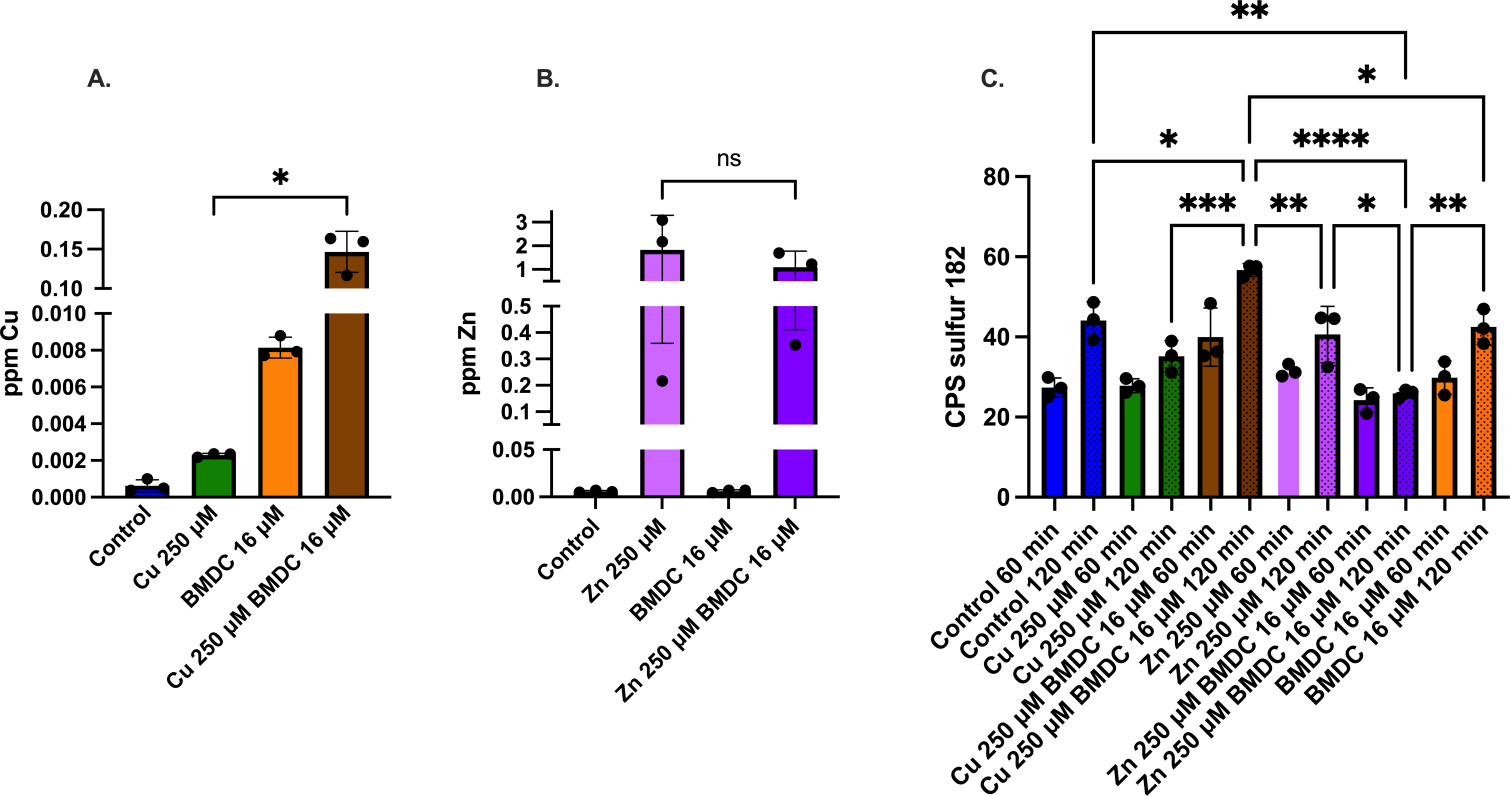
ICP-OES analysis of intracellular metal accumulation. MRSA was exposed to metal, BMDC, or a combination of metal-BMDC for 30 min. Following treatments, bacteria were washed with an EDTA buffer and lysed. ICPOES was used to determine the metal content associated with bacteria. (**A**) Intracellular copper concentration comparing copper against copper-BMDC treatment. (**B**) Intracellular zinc concentration comparing the statistical significance of zinc-BMDC against the zinc-only condition. (**C**) Sulfur values at indicated time points and treatments. Statistical differences were measured with an unpaired *t*-test with Welch’s correction (**A and B**) and a one-way ANOVA (**C**) with Tukey’s multiple comparisons test (ns, non-significant; *, *P* < 0.05; **, *P* < 0.01; ***, *P* < 0.001; ****, *P* < 0.0001).

Previously, we reported on the copper-DMDC action against *S. pneumoniae*, finding that the mechanism involved an increase in intracellular copper leading to copper intoxication (37). To determine if the same mechanism is happening in MRSA, we used Inductively Coupled Plasma Optical Emission Spectrometry (ICP-OES) to measure intracellular copper in the planktonic population after exposure to copper alone and in combination with BMDC. Copper levels were 70-fold higher at 30 min in the combination treatment compared to copper alone, indicating that BMDC also promoted an increase in intracellular copper in planktonic MRSA cultures (Fig. 2A). This accumulation continues for at least 120 min (Fig. S3A), where the concentration of copper reached twofold higher than with copper alone, suggesting a decrease in the rate of copper intake over time, an increase in the copper efflux, or both. Interestingly, there was no significant increase in zinc in the zinc vs the zinc-BMDC conditions, indicating that intoxication is not the primary mechanism of toxicity (Fig. 2B).

Our experiments also show that copper-BMDC or zinc-BMDC does not affect each other’s flux (Fig. S3A and B). However, copper with or without BMDC, and zinc-BMDC caused a statistically significant decrease in the manganese and magnesium pools (Fig. S3C and D). Yet, these reductions are minor when compared to the changes in the intracellular copper and zinc pools. Unfortunately, it is unclear if the changes in trace metal concentration are a consequence of mis-metalation by copper or zinc, a change in metal flux, or a consequence of metabolic changes within the bacteria.

As a component of amino acids, sulfur is essential to life and critical in the proteins that contain iron-sulfur clusters. Fenton chemistry oxidizes proteins, necessitating additional resources for living and repair pathways. Consequently, bacteria require an uptake of elements such as sulfur. Indeed, within 60 min, sulfur levels increase significantly in the copper-BMDC condition as compared to the control or copper-only conditions (Fig. 2C), indicating an abundance of sulfur associated with the cell. As sulfur is an integral component of BMDC (Fig. S1), we hypothesize that the mechanism of copper intoxication involves the uptake of copper-BMDC. As such, we observed that by 120 min, sulfur continues to increase independently of population growth across all conditions, particularly in the copper-BMDC combination, where it is 1.6-fold higher than in the copper-only condition. In contrast, sulfur did not increase in the zinc-BMDC combination, remaining unchanged from the control conditions, suggesting both a lack of BMDC internalization and cessation of metabolic activity.

**Fig 3:**
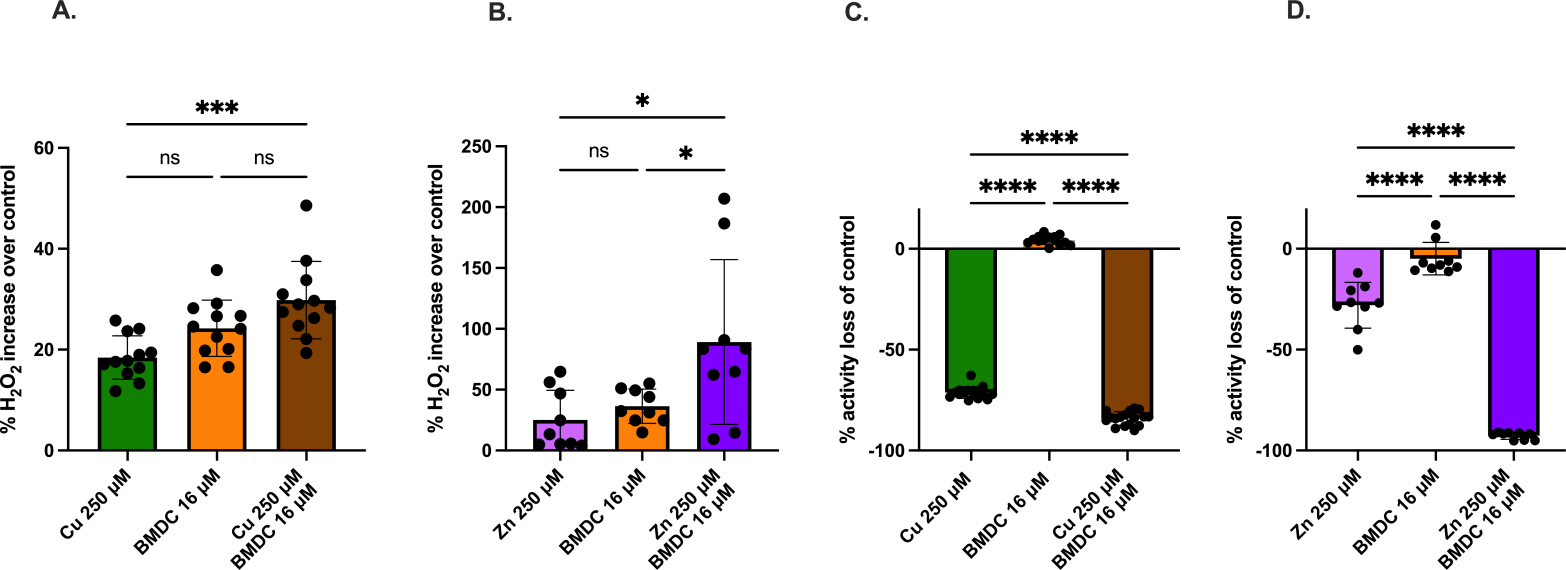
The metal-BMDC toxicity induces metabolic disruptions. MRSA was incubated for 1 h with the indicated treatments and an untreated control. (**A and B**) Peroxide production was assessed using the Pierce Peroxide Detection kit, with activity measured after 60 min of incubation, followed by 20 min of reagent development. (**C and D**) Resazurin-based metabolic assay performed after 60 min of treatment incubation, followed by 30 min of reagent development. Statistical differences were measured using a one-way ANOVA with Tukey’s multiple comparisons test (ns, non-significant; *, *P* < 0.05; **, *P* < 0.01; ***, *P* < 0.001; ****, *P* < 0.0001).

We next examined other common molecular processes disrupted by antibiotics and metal flux. In measuring peroxide accumulation 90 min after exposure, we found that copper or BMDC alone resulted in a 17% and 24% increase in peroxide levels, respectively, as compared to the control (Fig. 3A). Likewise, the copper-BMDC combination led to a 30% increase in peroxide levels (Fig. 3A). Similar to copper-BMDC treatments, zinc-BMDC treatment also significantly increases cellular peroxide levels approximately 100-fold over control (Fig. 3B)

We also investigated the metabolic activity of MRSA after treatment by using a resazurin assay. This assay relies on the reducing agent NADH to reduce resazurin to resorufin, resulting in a color change and an increase in fluorescence. Non-viable or metabolically impaired cells cannot reduce resazurin, leading to a lack of resorufin production and a decrease in absorbance. As expected, copper alone reduced metabolic output by 70%, whereas copper in combination with BMDC led to an 84% loss of metabolic activity (Fig. 3C). The copper-BMDC treatment resulted in a half-log decrease in CFU during the 90 min incubation period compared to the no-treatment condition (Fig. S4). This decrease was statistically significant, but it was unlikely to account for the full decrease in metabolic activity. Furthermore, zinc-BMDC treatments significantly decreased resazurin reduction as compared to the control, indicating impaired metabolic function (Fig. 3D), and the growth curves also support this observation (Fig. 1B).

**Fig 4:**
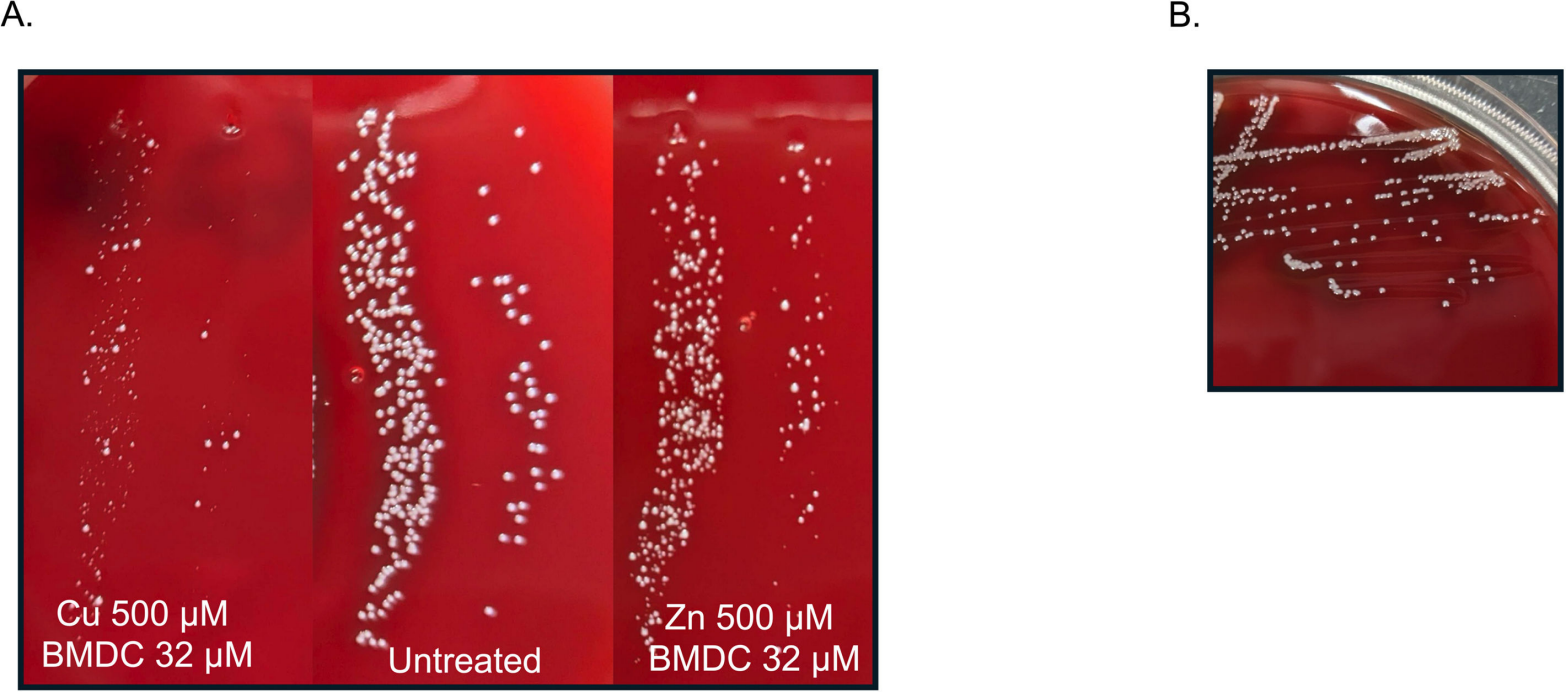
Metal-BMDC exposure of MRSA induces the formation of small colony variants. (**A**) Bacteria were exposed to 32 µM BMDC and either 500 µM copper or 500 µM zinc for 60 min. Samples were collected, diluted, and incubated for 17 h. Size comparison was performed against an untreated parallel culture. (**B**) A small colony variant was collected with an inoculating loop and streaked onto a new plate; size was verified 12 h later.

Different stressful environments can lead to bacterial adaptation. One way bacteria cope with these stresses is by forming small colony variants (SCVs), which typically involve the bacteria reducing their metabolism and slowing growth (39, 40). Exposure to sublethal amounts of BMDC in combination with either copper or zinc led to the formation of SCVs, demonstrating that this treatment induces metabolic perturbations. However, subculturing the SCVs on blood agar plates restored them to their normal growth phenotype, demonstrating that the variant phenotype is reversible upon removal of copper-BMDC or zinc-BMDC stress (Fig. 4A and B).

### Metal-BMDC disrupts biofilm formation and promotes clearance of mature MRSA biofilm

**Fig 5:**
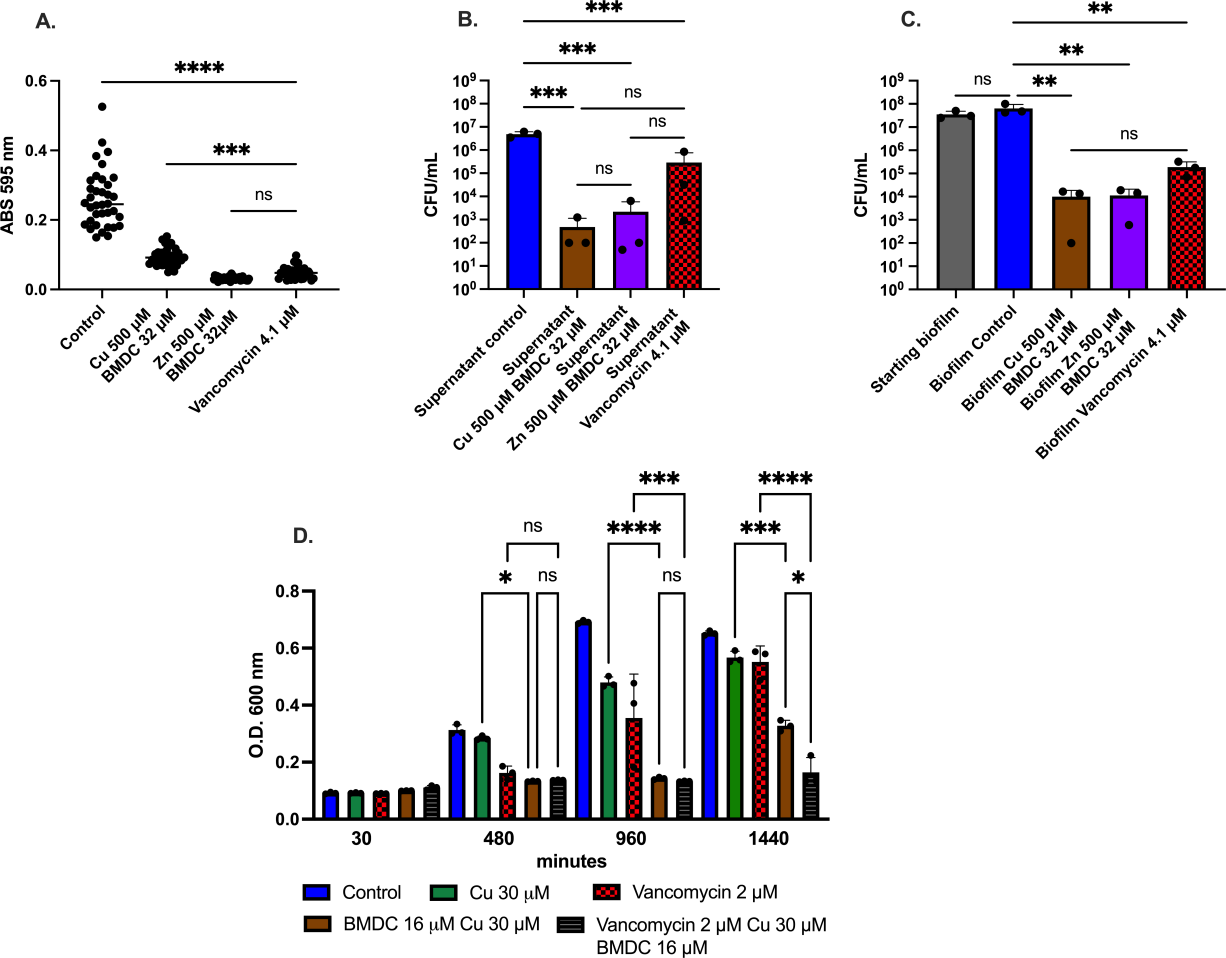
MRSA viability and biofilm after metal-BMDC combination or vancomycin exposure. Biofilms were allowed to form for 24 h, after which non-adherent cells were removed. Wells were then treated with either metal-compound or vancomycin. (**A**) Biofilm quantified using the crystal violet assay. (**B**) Viable bacterial counts from the supernatant. (**C**) Viable bacterial counts from the adherent biofilm population following washing. (**D**) Representative timepoints taken from a growth curve showing combination treatment with vancomycin for 24 h. Statistical differences were measured with a one-way ANOVA with Tukey’s multiple comparisons test (*, *P* < 0.05; **, *P* < 0.01; ***, *P* < 0.001; ****, *P* < 0.0001).

After establishing that BMDC, combined with either copper or zinc, has antimicrobial activity against planktonic bacteria, we tested it against cells growing in biofilms. We utilized the frequently used crystal violet assay as a proxy for biomass produced by bacteria after treatments (41–44). Biofilm production was further confirmed by staining biofilm with safranin (Fig. S5). While using BMDC with copper or zinc decreased the overall ability to form biofilms due to bacteriostatic effects, we allowed bacteria to establish over 24 h to determine the treatment effects on mature biofilms. There was a statistically significant decrease in the amount of biomass (as determined by crystal violet staining) retained by the biofilm when treated with BMDC in combination with either copper or zinc (Fig. 5A). This decrease correlated with a significant reduction in the colony counts recovered from either the supernatant or the biofilm population (Fig. 5B and C). These results demonstrate that BMDC sensitizes MRSA to copper intoxication, reduces planktonic and biofilm-associated bacteria, and decreases overall biofilm *in vitro*.

### Enhancing vancomycin efficacy through copper-BMDC combinatory treatment

A treatment for invasive MRSA infections is intravenous vancomycin. The broth microdilution method revealed that 4.1 µM (6 µg/mL) of vancomycin had bacteriostatic effects for 40 h, whereas 2 µM was subinhibitory. As chronic vancomycin usage can be toxic to the kidneys (45, 46), we investigated whether adding copper-BMDC could restore vancomycin susceptibility at subinhibitory concentrations. We selected a physiologically relevant concentration of copper that reflects levels found across various human anatomical sites (not just within the phagolysosome of macrophages which can be above 500 µM), both during infected and non-infected conditions (47, 48). In growth curves, a combination of 30 µM copper, 16 µM BMDC, and 2 µM vancomycin produced bacteriostatic effects for nearly 24 h, demonstrating potential for combinatory treatment that relies on copper-BMDC and vancomycin (Fig. 5D).

Using an *in vitro* biofilm model, we evaluated the efficacy of BMDC combined with either copper or zinc and compared it against the clinically relevant antibiotic vancomycin. This treatment was as effective as vancomycin in reducing biofilm biomass, showing no statistical difference between the metal-BMDC combination and vancomycin (Fig. 5A). Additionally, colony-forming units collected from both the supernatant and the biofilm-associated population revealed a significant decrease in viable bacterial counts when compared to untreated controls. This indicates bactericidal activity against both planktonic and biofilm-associated bacteria (Fig. 5B and C), with no statistically significant difference between treatments.

### Biofilm dynamics during copper-dependent treatment

**Fig 6:**
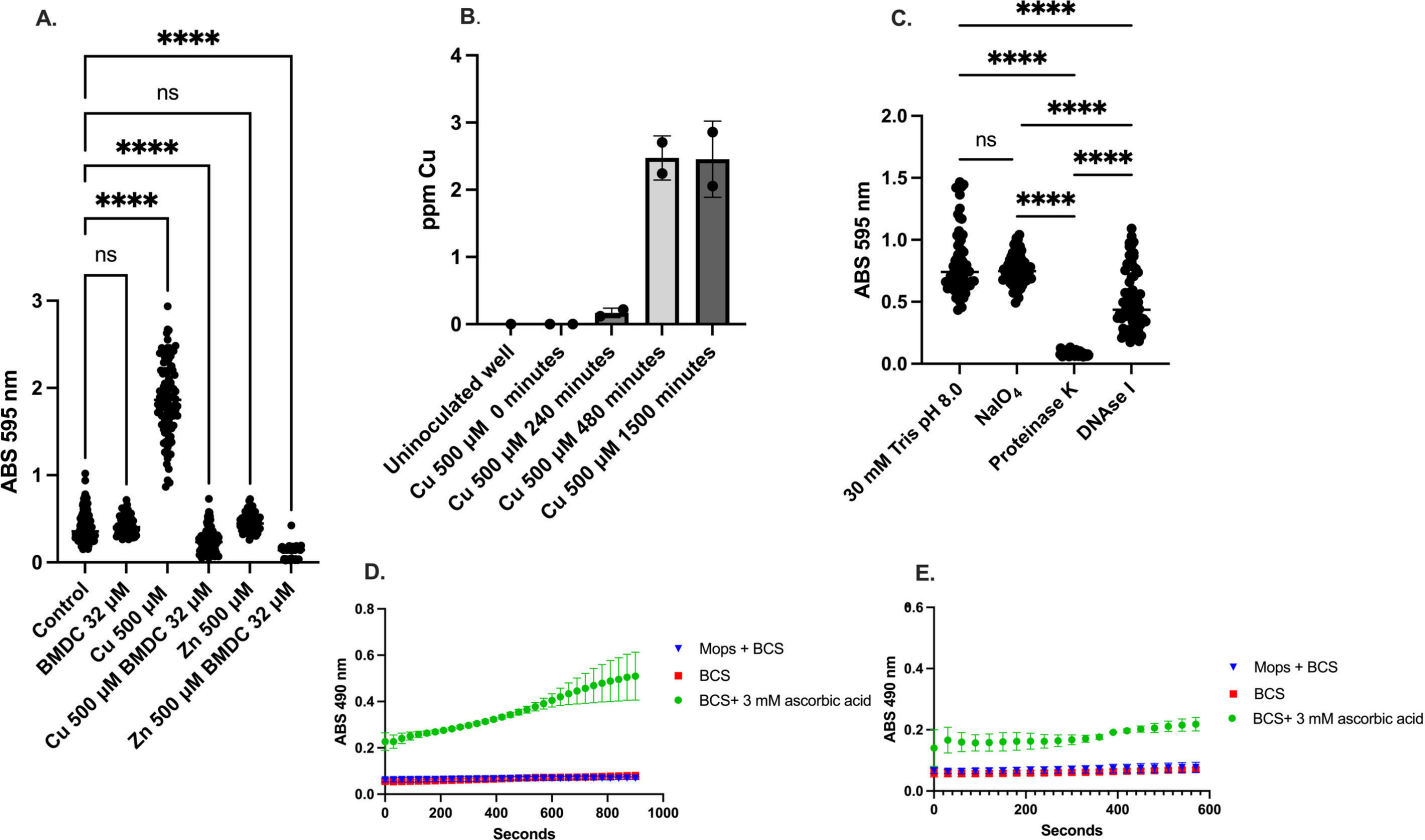
Copper increases biofilm production when exposed without BMDC. (**A**) Crystal violet stain showing biofilm accumulation after the denoted treatments. (**B**) Copper content as analyzed by ICP-OES from biofilm after incubation with 500 µM copper for the specified time. (**C**) Crystal violet staining of biofilm after incubation with 500 µM of copper for 24 h, followed by a wash period and incubation with the specified biofilm disruptors. Tris buffer (control), sodium periodate (polysaccharides), proteinase K (proteins), Dnase I (eDNA). (**D**) Bathocuproinedisulfonic acid assay showing copper accumulation in established biofilms after copper exposure and washout period. Samples were taken at 24 h, and ascorbic acid was used as a reductant during the assay. (**E**) Same as D, but taken at 8 h, ascorbic acid was used as a reductant during the BCS assay. Statistical differences were measured by a one-way ANOVA with Dunnett’s multiple comparisons test (**A**) and Tukey’s multiple comparisons test (**C**) (ns, non-significant, *, *P* < 0.05; **, *P* < 0.01; ***, *P* < 0.001; ****, *P* < 0.0001).

Interestingly, copper alone increased the production of biofilm (Fig. 6A) when compared to the control condition, and this was accompanied by an increase in copper content in the biofilm, which reached saturation at 480 min (Fig. 6B). Further investigation of the biofilm structure suggested that this increase was primarily associated with protein production, as treating the biofilm with proteinase K significantly decreased the amount of recoverable biofilm as compared to using DNAse, to degrade DNA, and NaIO_4_, to disrupt carbohydrates (Fig. 6C). Furthermore, BMDC still sensitized bacteria to metal stress in the biofilm even after 6 h of metal-only exposure, as demonstrated by the decrease in CFU compared to the control, as well as an increase in copper accumulation in the biofilm (Fig. S6A through C). To confirm that the biofilm retained copper, we employed the Cu^1+^ chelator bathocuproinedisulfonic acid (BCS) assay (49). Copper was added to the biofilm, and samples were taken at either 8 or 24 h after removal of non-biofilm-associated copper. Following the addition of ascorbic acid, copper was reduced and reacted with BCS, resulting in an absorbance that was measured at 490 nm. Longer incubations with copper in the biofilm resulted in a larger increase in BCS signal (Fig. 6D) compared to shorter incubations with copper (Fig. 6E), indicating that copper absorption into the biofilm is time-dependent and requires the presence of biofilm (Fig. 6B). Likewise, this confirmed that in the biofilm, the oxidation state of copper is Cu^2+^ (Fig. 6D and E).

**Fig 7:**
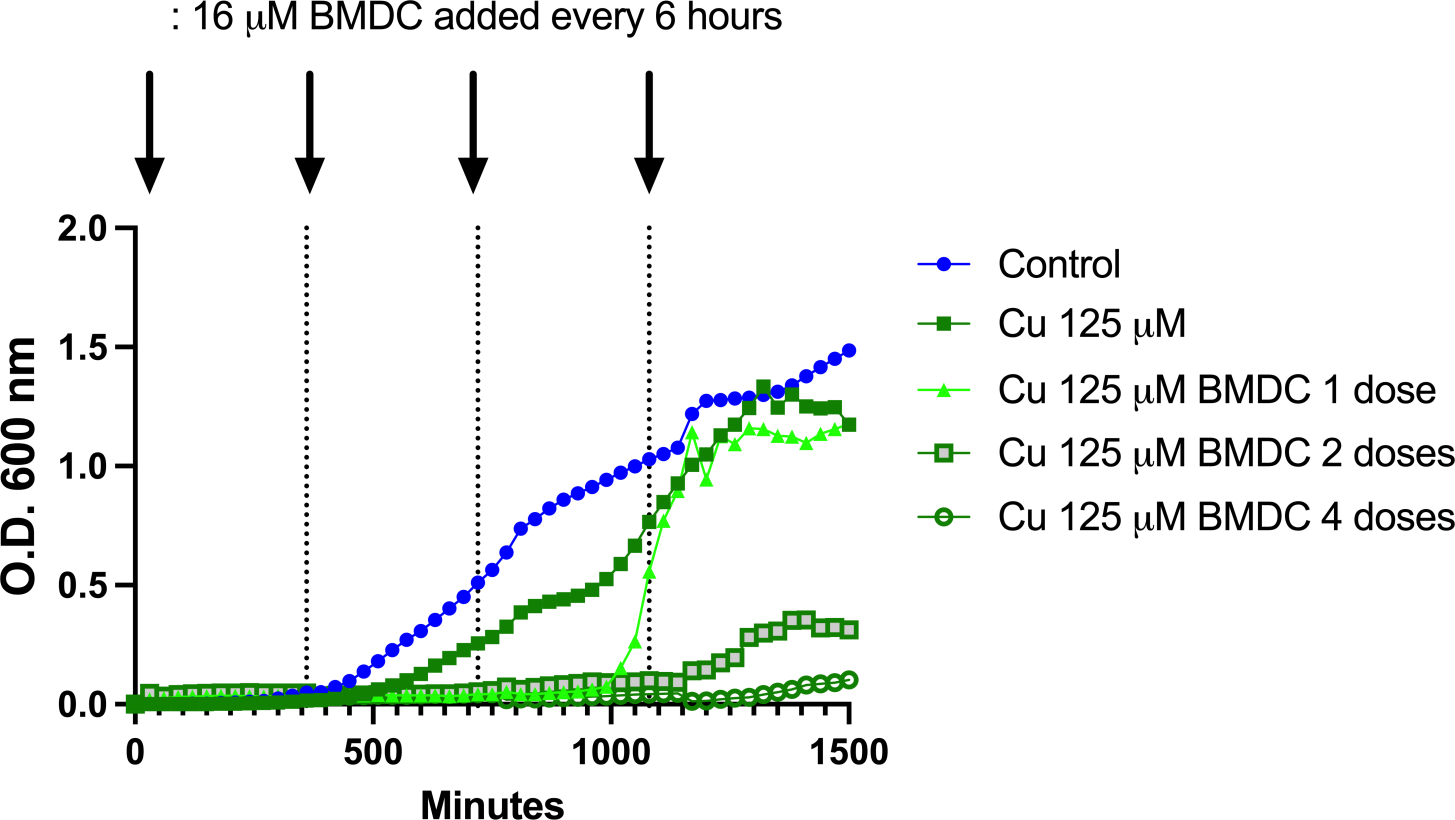
Sequential doses of BMDC sensitize bacteria in biofilm to copper toxicity. Biofilm was established over 24 h, followed by washing to remove non-adherent cells. Fresh media with 125 µM copper was added, and BMDC 16 µM was administered every 6 h as denoted by the arrows. Optical density was monitored for 24 h to assess treatment efficacy and biofilm dynamics.

To explore the dynamics affecting biofilm formation after metal-compound treatment, we used sublethal combinations of metal and compound to treat the biofilm. Biofilms undergo distinct developmental stages, including initial attachment and colonization of a surface, establishment of the biofilm, maturation, and dispersal (50). After allowing the biofilm to develop for 24 h, the wells were washed and supplied with either fresh medium or fresh medium containing sub-inhibitory concentrations of copper and BMDC. There was an increase in the optical density of the untreated control 6–8 h after washing, which continued for the remainder of the experiment. However, the wells containing 125 µM of copper, in addition to having 16 µM of BMDC added every 6 h for 18 h, maintained their bacteriostatic effects for 24 h (Fig. 7). We hypothesized that as bacteria in the biofilm disperse, they become more susceptible to the effects of copper and BMDC. Figure 5B supports this hypothesis, as most bacterial counts remained below the detection limit.

### Antimicrobial and antibiofilm activity of metal-BMDC against *Staphylococcus epidermidis*

**Fig 8:**
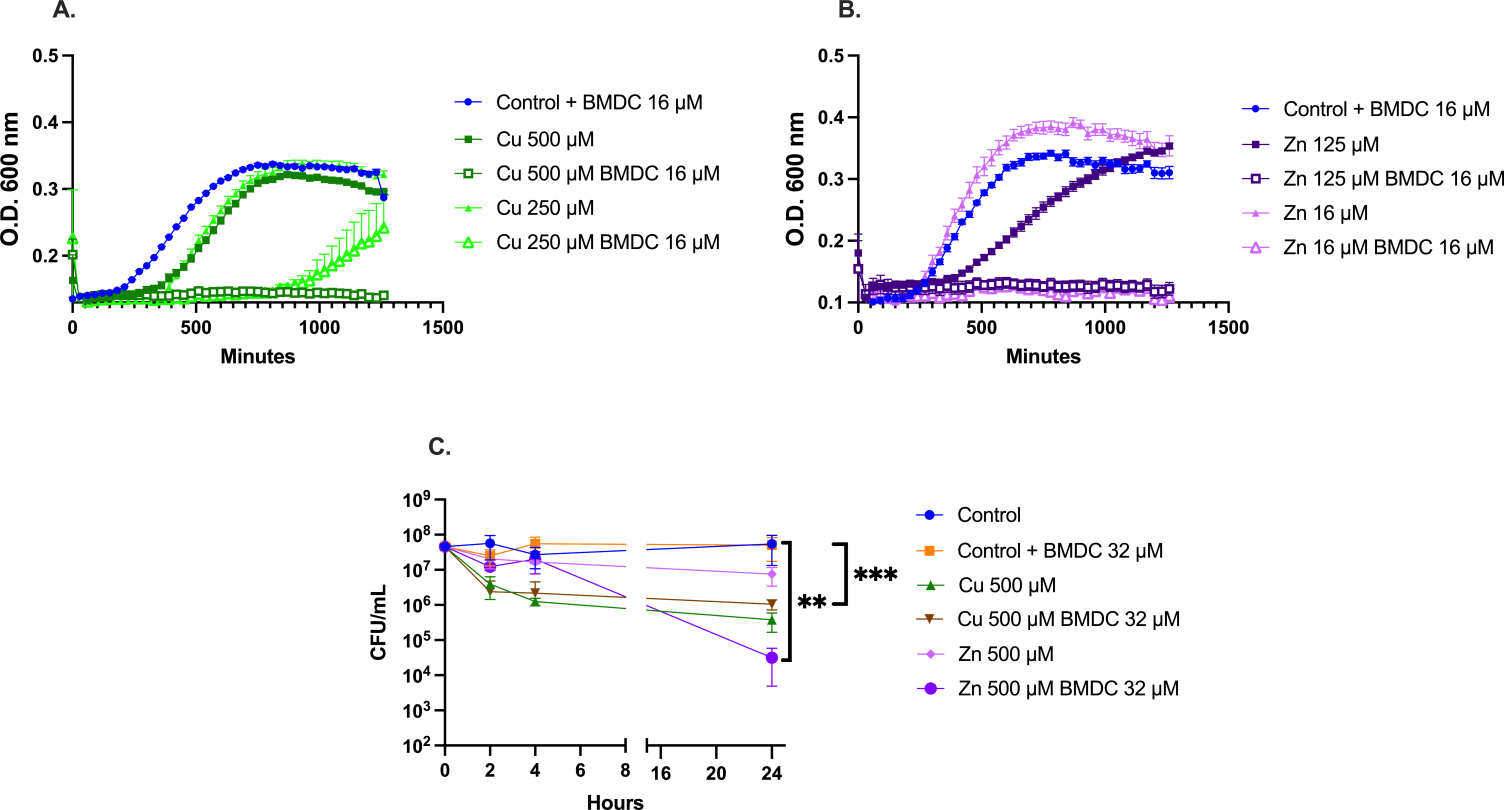
BMDC sensitizes *Staphylococcus epidermidis* to copper and zinc toxicity. (**A and B**) Growth curve showing bacterial growth with or without BMDC during copper or zinc exposure. (**C**) Survival assay showing CFU recovered at indicated time points following treatments. For (**C**), statistical differences were calculated using AUC followed by an ordinary one-way ANOVA with Tukey’s multiple comparisons test (ns, non-significant, *, *P* < 0.05; **, *P* < 0.01; ***, *P* < 0.001; ****, *P* < 0.0001).

**Fig 9:**
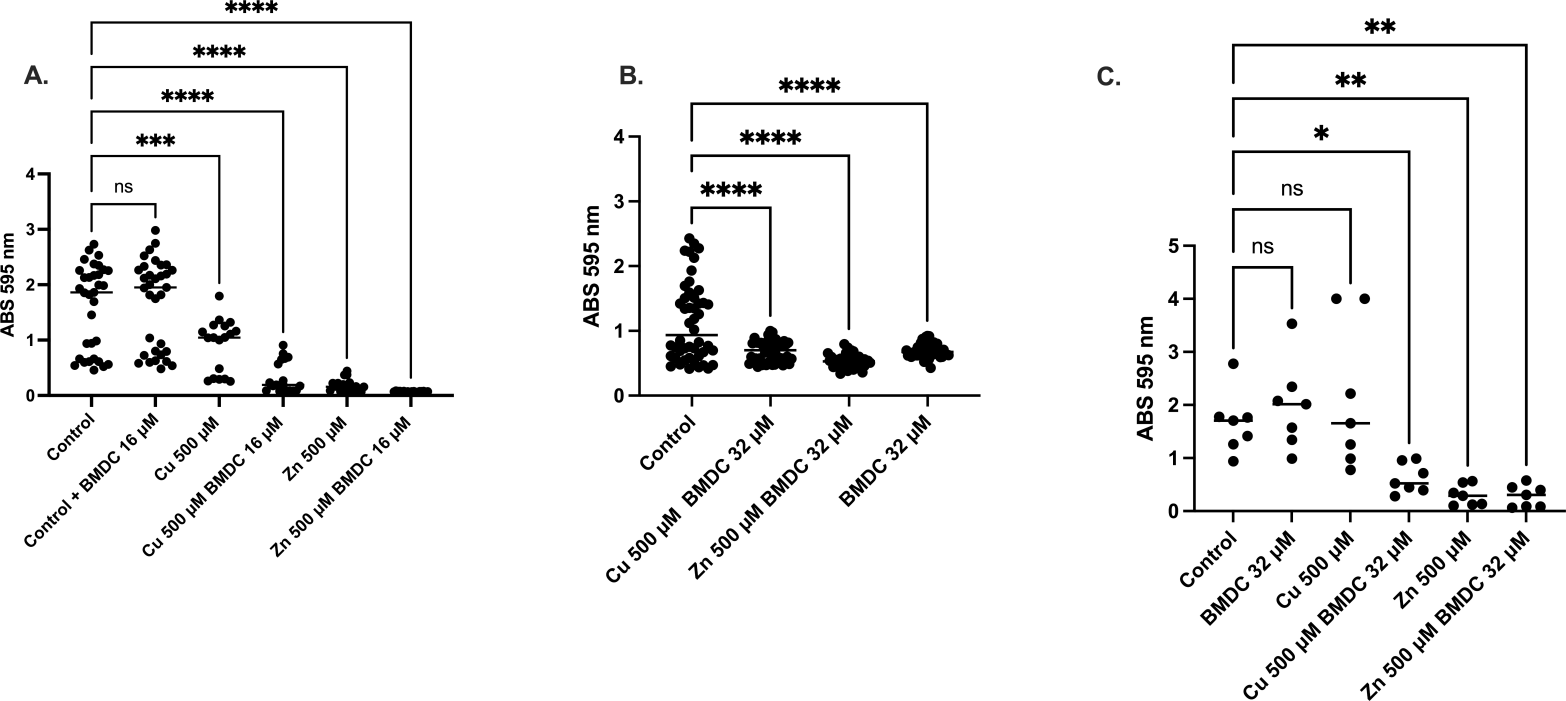
Crystal violet assay representing biofilm biomass after 24 h of incubation with indicated treatments. (**A**) Crystal violet stain measured after 24 h of a growth curve experiment. (**B**) Crystal violet stain of an established biofilm after treatments. Biofilm was allowed to establish for 24 h; non-adherent cells were washed, and new medium with treatments was supplied. Crystal violet was used to stain the remaining biofilm. (**C**) Biofilm determination in ultra-high-molecular-weight polyethylene tape. Statistical differences were measured using a one-way ANOVA with Dunnett’s multiple comparisons test (ns, non-significant, *, *P* < 0.05; **, *P* < 0.01; ***, *P* < 0.001; ****, *P* < 0.0001).

*S. epidermidis* is another clinically relevant species known to form biofilm. Akin to its effects on MRSA, BMDC was effective in sensitizing *S. epidermidis* to copper intoxication (Fig. 8A). Furthermore, *S. epidermidis* exhibited comparable susceptibility to zinc-BMDC as observed in MRSA, with growth inhibition occurring at all tested concentrations (Fig. 8B). Survival assays revealed bactericidal and bacteriostatic effects over 24 h, with copper-BMDC showing similar survival rates to those of copper alone. In contrast, zinc-BMDC exhibits bactericidal activity over 24 h, suggesting a mechanism of action resembling that observed against MRSA (Fig. 8C). Furthermore, metal-BMDC combinations reduced biofilm formation as indicated by a decrease in crystal violet staining (Fig. 9A through C). Surprisingly, BMDC 32 µM alone had antibiofilm activity when compared to the untreated control (Fig. 9B). In contrast to MRSA, copper alone did not enhance the biofilm production of *S. epidermidis* (Fig. 9A and C); however, the metal-BMDC combination was equally effective at decreasing an established biofilm *in vitro* (Fig. 9B).

Finally, because *S. epidermidis* is a leading cause of prosthetic joint infections, we sought to evaluate the potential of metal-BMDC-assisted biofilm eradication on relevant surfaces. To approximate the material properties of prosthetic implants, we used ultra-high molecular weight polyethylene tape as a model substrate and conducted biofilm assays. As with the plate assays (Fig. 9A and B), copper-BMDC and zinc-BMDC were able to disrupt biofilm formed on the surface of the ultra-high molecular weight polyethylene (Fig. 9C), demonstrating clearance of biofilm on two different surfaces for *S. epidermidis* (Fig. 9B and C).

## DISCUSSION

We evaluated the antimicrobial and antibiofilm properties of metal-BMDC against Staphylococcal species, including MRSA and *S. epidermidis*. Growth curves and survival assays revealed bactericidal and bacteriostatic effects induced by the metal-BMDC combinations, with activity sustained for 24 h. BMDC combined with copper resulted in concentration-dependent growth inhibition, including an 8–10 h growth delay at lower concentrations. In contrast, zinc-BMDC produced a distinct bactericidal effect, characterized by a failure to proliferate that persisted for at least 48 h. Furthermore, survival assays demonstrated that copper-BMDC treatment caused a rapid decline in bacterial viability leading to bacteriostatic effects. Meanwhile, zinc-BMDC exposure led to a slower decline in bacterial viability, resulting in bactericidal effects. These findings suggest that although both treatments disrupt bacterial physiology, they do so via different mechanisms; copper-BMDC exerts immediate stress that bacteria can partially adapt to, while zinc-BMDC appears to target a critical pathway, preventing adaptation and enabling antimicrobial activity.

Further evidence supporting the distinct mechanisms of metal-BMDC can be elucidated from ICP-OES analysis. BMDC, functioning as a metal-dependent antimicrobial, significantly enhanced intracellular copper accumulation—reaching a 70-fold increase within 30 min of exposure and continued to rise over 2 h, likely leading to copper intoxication. In contrast, zinc levels during zinc-BMDC treatment remained unchanged, indicating that BMDC did not enhance zinc uptake and, thus, is unlikely to cause zinc toxicity. This divergence in metal accumulation profiles underscores the mechanistic differences between copper-BMDC and zinc-BMDC treatments.

Elevated copper uptake correlated with potent activity against both planktonic MRSA and cells within mature biofilms, suggesting that copper intoxication is a key mechanism of action. Copper intoxication in bacteria operates through multiple damaging mechanisms, and we found evidence to support several of them. One key mechanism is mis-metalation, where copper displaces native metal cofactors in proteins (51). This disrupts the function of critical enzymes, including redox sensors and iron-sulfur containing proteins (52, 53). Another mechanism is Fenton chemistry, in which copper catalyzes the formation of ROS. Consistent with these mechanisms, our data show a 30% increase in peroxide production within 90 min of copper-BMDC exposure compared to control conditions. These results align with a previous microarray analysis of the *S. aureus* Newman strain, which revealed a transcriptional signature of oxidative stress following copper exposure (54). Likewise, zinc-BMDC exposure led to a roughly 100% increase in peroxide accumulation; still, the mechanism underlying this increase in peroxide remains under investigation. However, previous literature on *S. pneumoniae* has shown that exposure to excess zinc disrupts manganese transport, with an EC_50_ of 30 µM, resulting in damaged oxidative relief pathways (55).

Manganese is a critical cofactor for superoxide dismutase (SOD), an enzyme that protects bacteria from oxidative stress by neutralizing ROS. Previous research has demonstrated that manganese supplementation enhances SOD activity, thereby improving bacterial resistance to oxidative agents such as methyl viologen (56). In our study, we observed a statistically significant decline in intracellular manganese levels 120 min after exposure to metal-BMDC treatment. This depletion could compromise SOD function, thereby reducing the bacteria’s ability to detoxify ROS. As a result, this could explain the elevated peroxide accumulation seen in the treated MRSA population.

Peroxide accumulation has harmful effects in bacteria and can lead to metabolic disruption, as supported by our resazurin assay. Following copper-BMDC treatments, resorufin production decreased by 84%, indicating a significant impairment in metabolic activity. Since the assay relies on NADH availability and dehydrogenase function, this reduction reflects a perturbed redox balance and compromised energy metabolism. Prior literature including a proteomic analysis of the *S. aureus* SH1000 strain revealed a significant decrease in lactate dehydrogenase protein abundance after exposure to 1.5 mM copper, suggesting that copper interferes with key metabolic pathways required for energy metabolism (57). Zinc-BMDC treatment was equally detrimental to bacterial metabolism, causing a 90% reduction in resorufin production. However, unlike copper, this effect is unlikely to be driven by zinc intoxication, as ICP-OES analysis revealed that intracellular zinc levels remained unchanged regardless of the presence of BMDC (Fig. 2B). Moreover, the zinc-BMDC treatment exhibited a threefold decrease in metabolic activity compared to treatment with zinc alone (Fig. 3D). These findings suggest that metabolic impairment likely contributes to the antimicrobial effects of metal-BMDC against MRSA.

Exposure to the metal-BMDC combination led to the emergence of small colony variants (SCV), a phenotype associated with metabolic adaptations. SCV can arise in response to oxidative stress, ATP depletion, and reduced membrane potential (39, 58, 59). In our study, the reversible nature of SCV formation (demonstrated by restored growth upon subculturing on blood agar plates) suggests that these variants result from transient metabolic stress rather than permanent genetic changes (Fig. 4B). Importantly, these SCVs were unculturable after 24 h of continuous exposure, indicating that even if MRSA shifts to this variant, it remains susceptible to being killed by the metal-BMDC treatment.

Metal-BMDC treatment significantly reduced established biofilms. Copper-BMDC decreased biofilm by 64%, while zinc-BMDC or vancomycin reduced biofilm by 87% and 81%, respectively. Bacterial counts in both the biofilm structure and the supernatant were significantly reduced by 3–4 logs, indicating a strong antimicrobial activity with distinct susceptibilities to metal-BMDC treatment. These differences likely reflect different mechanisms of toxicity, as our data suggest. While BMDC sensitizes bacteria to copper-induced toxicity, the mechanism of zinc-BMDC toxicity is the subject of ongoing investigations. Our findings indicate that copper-BMDC enters the bacteria, as evidenced by an early increase in copper and sulfur concentration. In contrast zinc-BMDC does not increase zinc levels beyond those in zinc-only conditions and does not increase internal sulfur content, suggesting that the complex does not enter the bacterial cell and likely targets something in the membrane.

BMDC enhanced copper sensitivity in both *S. epidermidis* and MRSA, resulting in bactericidal and bacteriostatic effects in planktonic cultures, as well as a progressive reduction in biofilm. Surprisingly, the copper-only condition increased biofilm production in MRSA, without affecting bacterial counts, and did not affect the biofilm of *S. epidermidis*. Increases in biofilm have been observed after exposure to tobramycin, amikacin, and streptomycin in *Pseudomonas aeruginosa* (60). Knockouts in exopolysaccharide production of this bacterium retained similar biofilm production after tobramycin exposure, indicating that this biomass was not primarily composed of this polysaccharide. However, colony counts indicated an increase in the *P. aeruginosa* population. In contrast, our experiment showed that colony counts did not increase, suggesting that copper-induced biofilm formation is a stress response rather than a growth-related phenomenon. When gradually intoxicated with copper, MRSA adapted by increasing biofilm components via proteins and eDNA (Fig. 6C). This result suggests that MRSA infections could be exacerbated by insufficient dosage or duration of treatment. We hypothesize that the protein component in the copper-induced biofilm includes extracellular matrix protein (EMP), which facilitates cellular aggregation in biofilms (61). This protein has been identified in highly invasive Staphylococcal species, such as *S. aureus* and *S. haemolyticus;* however, bioinformatics data (62) suggest that it is absent in low-pathogenic species such as *S. epidermidis*. Sequence analysis of EMP reveals multiple histidine and tyrosine residues, which could facilitate copper binding, thus acting as a copper sponge. Attempts to model the EMP-copper interaction using AlphaFold were unsuccessful, as the protein appears to be highly disordered.

The immune system mobilizes both copper and zinc during infection. We have demonstrated that the combination of these metals, aided by BMDC, becomes robust antibacterial and antibiofilm agents, achieved through multiple target disruptions in the bacterial cell. Copper-BMDC promotes mis-metalation, redox imbalances, and metabolic perturbations, while zinc-BMDC causes toxicity through a mechanism that remains to be explored. Building on our previous findings that BMDC reduces bacterial burden in a murine lung infection model via inhalation (38), we now demonstrate its efficacy in additional settings relevant to human health. Most pertinent is BMDC’s clearance of biofilms on ultra-high molecular weight polyethylene, a material vital for knee, hip, and other joint replacement surgeries due to its low immunogenicity and high wear resistance. A future direction is to investigate the effects of metal-BMDC-specific pathogen clearance on additional biological surfaces. Together, these combinations exert potent bactericidal effects against planktonic MRSA and *S. epidermidis,* while also effectively disrupting and eradicating established biofilms, highlighting their potential as a powerful antimicrobial strategy.

## MATERIALS AND METHODS

### Synthesis of *N*-benzyl-*N*-methyldithiocarbamate

A solution of sodium hydroxide (1.00 mmol) in ethanol (10 mL) was cooled to 0°C and stirred for 5 min. Then, *N*-methylbenzylamine (130 µL,1.00 mmol) and carbon disulfide (151 µL, 2.50 mmol) were added to the solution successively. The resulting mixture was stirred at room temperature for 2 h, after which the solvent was reduced under vacuum. Sodium *N*-benzyl-*N*-methyldithiocarbamate (BMDC) was synthesized with a 68% yield as a white solid. ^1^H NMR (400 MHz, DMSO-*d*6) δ 7.28–7.19 (m, 4H), 7.19–7.12 (m, 1H), 5.43 (s, 2H), 3.24 (s, 3H). ^13^C NMR (100 MHz, DMSO-*d*6) δ 215.70, 139.58, 128.42, 127.66, 126.75, 57.85, 40.99. *T*_decomp_. = 228–229°C (capillary, no melting).

### Cell lines and growth conditions

Methicillin-resistant *Staphylococcus aureus* ATCC 33591 was cultured on either Tryptic Soy Agar plates with 5% sheep’s blood with neomycin or Mannitol Salt Agar (MSA) plates overnight at 37°C with 5% CO_2_ supplementation. *Staphylococcus epidermidis* was kindly donated by the University of Arizona Microbiology Core and was cultured on MSA plates or BAP without antibiotics, in the same manner as MRSA. A bacterial sample was used to inoculate RPMI 1640 supplemented as per (63) with the following added trace metals: 246 µM MgCl_2_.6H_2_O, 5.3 µM CaCl_2_.2H_2_O, 179 nM FeSO_4_.7H_2_0, 200 nM CuSO_4_.5H_2_O, 173 nM ZnSO_4_.7H_2_O, and 87 nM MnCl_2_ to create an inoculum equivalent to 10^6–10^7 CFU/mL.

### Growth curves

Bacterial aliquots were thawed and diluted 1:10 with modified RPMI (as described above). Treatments were added to the wells and serially diluted to reach an appropriate concentration in 96-well Greiner Bio-One polystyrene plates. Growth curves were monitored using a Biotek Cytation5 at 37°C, 5% CO_2_, and relative humidity, with optical density measurements taken every 30 min.

### Survival assays

Bacterial aliquots were thawed and diluted to 1:10 in modified RPMI 1640. The culture was divided into the indicated treatments or control groups. Bacteria were incubated at 37°C with 5% CO_2_ supplementation for the appropriate times. Representative samples were collected, serially diluted, and plated onto Tryptic Soy Blood agar (as Mannitol Salt agar plates were found to be detrimental after our treatment) to determine viability. Plates were incubated for up to 60 h to evaluate the absence of growth. When colonies were not detected, the dilution series was labeled as 50 CFU/mL to account for the technical limitations of a dilution series.

### Biofilm experiments

For the biofilm experiments, bacterial aliquots were thawed, added to fresh media, and distributed into a 96-well flat-bottom plate (Greiner Bio-One Cellstar). The plates were incubated statically at 37°C under 5% CO₂ for 22–24 h. After incubation, the supernatant was removed, and the plates were washed twice with ultrapure water. The plates were replenished with fresh, warm media and treatments, followed by an additional 22–24 h of incubation. At the end of this period, the supernatant was collected to assess viability. The plates were washed twice with ultrapure water and allowed to dry overnight.

### Bacterial viability in biofilm

Ninety-six-well plates were seeded as described above. After a 24 h incubation, the supernatant was removed, the plates were washed with ultra-pure water, and the wells were replenished with either fresh medium or a combination of medium with 500 µM copper and/or 32 µM of BMDC. The plates were incubated for an additional 24 h. At the end of the incubation period, the supernatant was collected and combined to a total volume of 1 mL. The wells were rinsed with sterile PBS, followed by the addition of 100 µL of PBS. A sterile pipette tip was then used to scrape the bottom of the wells until clear. Pooled PBS from scraped wells was serially diluted and plated onto BAP to determine bacterial viability from either the supernatant or scraped population (considered biofilm).

### Biofilm quantification assay

Overnight-dried plates were stained with 165 µL of 0.1% crystal violet solution in 0.025% methanol for 40 min, or 0.25% safranin in 9% Ethanol. Each plate had all conditions included to serve as their own control for staining. Excess dye was rinsed by adding 200 µL of ultrapure water (18.1 mW) twice for 2 min and allowing it to dry for at least 30 min. Destaining was achieved by adding 150 µL of a 30% acetic acid solution for 20 min at room temperature. One hundred microliters of this solution was transferred to a new plate and read at 595 nm on a Cytation 5. All statistical analyses were performed using the software GraphPad Prism 10.

### Ultra-high molecular weight polyethylene tape biofilm assay

Pieces of UHMWP tape were cut into 1 cm^2^ pieces and sterilized using high-pressure heat. These pieces were aseptically attached to the bottom of 12-well polystyrene plates and treated in the same manner as the 96-well biofilm assays. With the exception that after crystal violet staining and overnight drying, the pieces of tape were detached from the plate and added to 1 mL Eppendorf tubes, followed by the addition of 30% acetic acid to solubilize the dye. One hundred microliters of the solubilized sample was used to determine the absorbance at 595 nm.

### Biofilm disruption assay

After overnight drying of biofilm plates, they were subjected to the biofilm disruptors: 10 mM NaIO_4_ (prepared in either 100 mM Sodium acetate pH 4.5 or 20 mM TRIS pH 7.5), 100 µg/mL proteinase K in 20 mM TRIS pH 7.5, 100 µg/mL DNase I (prepared in either 150 mM NaCl, 1 mM CaCl_2_ and 2.5 mM MgCl2, or 20 mM TRIS pH 7.5), and 20 mM TRIS pH 7.5 buffer alone. Incubate at 37°C for 24 h and then wash and dry the plates. Stain the plates with 0.1% crystal violet to determine biomass as previously stated.

### Metal quantification via ICP-OES

*S. aureus* was grown to an equivalent 10^7 colony-forming units; these cultures were separated into control and treatment groups and incubated for 30 min statically at 37°C. Liquid bacterial samples were placed in an ice-water bath for 5 min, followed by centrifugation at 3,500 × *g* for 7 min at 4°C. This was followed by two washes with cold buffer (200 mM EDTA, 50 mM Tris pH 7.4, 150 mM NaCl). The supernatant was discarded, and 70% nitric acid was added, followed by overnight incubation at 65°C to digest the samples. Finally, ultrapure water was added to resuspend samples to a 2.5% acid concentration. The metal content was analyzed by means of an iCAP PRO XDUO ICP-OES with a wavelength of 324.754 nm for copper, 213.856 nm for zinc, 259.373 nm for manganese, 279.553 nm for magnesium, and 182.034 nm for sulfur. Standards were made using a multi-element standard from Inorganic Ventures, and the metal content of the bacterial samples was calculated using the Qtegra software.

### Metabolism assessment

Metabolic activity was determined using the resazurin assay (64–66). First, we optimized the concentration, cell density, and incubation times required to obtain a reliable signal (data not shown). MRSA was grown to an optical density of 0.3, equivalent to 10^7 CFU/mL. At this cell density, the culture was divided into six treatments: 250 µM copper or zinc, 16 µM BMDC, copper-BMDC or zinc-BMDC, and a control, followed by 60 min of static incubation at 37°C under 5% CO_2_. Then, the cultures were supplied with 50 µM resazurin and incubated for an additional 30 min. One hundred microliters of this reaction was immediately placed onto a Greiner Bio-One black-side, transparent-bottom 96 microtiter plate and read in a Biotek Cytation 5 with 540 nm excitation and 590 nm emission wavelengths, as well as absorbance at 590 nm.

### Hydrogen peroxide detection assay

Bacteria were grown as described in the metabolism assay. After the 60 min incubation, 20 mL of the sample was added to 80 mL of the working reagent of the Pierce Quantitative Peroxide Assay (Thermo Fisher). The mixture was covered in foil and incubated at room temperature for 20 min. Absorbance was read immediately at 595 nm, and values were recorded. Peroxide concentration was determined using a standard curve.

### Bathocuproinedisulfonic acid assay

Biofilms were prepared as before, washed, and dried overnight. One hundred millimolar BCS was prepared in a 10 mM MOPS buffer. A 3 mM ascorbic acid solution was prepared in MOPS buffer. One hundred microliters of MOPS, 1 mM BCS with or without 30 nM ascorbic acid was added directly to the biofilm that had been collected at specific times, and absorbance was read at 490 nm for 15 min in a Cytation 5.

### Metal quantification in biofilm via ICP-OES

Biofilms were prepared as previously, washed with ultrapure water, and dried overnight. One hundred microliters of 70% nitric acid was added directly to the wells and incubated at room temperature for 2 min. A pipette tip was used to scrape the bottom of the well, and 500 µl of these samples was combined and diluted with ultrapure water to 2.5% for ICP-OES analysis.

### Statistical analysis

All statistics were performed with the software GraphPad Prism 10 Version 10.6.0. Specific tests used for analysis are described in the figure legends.

## SUPPLEMENTAL MATERIAL

Supplemental figures msphere.00691-25-s0001.pdf Figures S1-S6.

ASM does not own the copyrights to Supplemental Material that may be linked to, or accessed through, an article. The authors have granted ASM a non-exclusive, world-wide license to publish the Supplemental Material files. Please contact the corresponding author directly for reuse.
