# Supplementary material for: N-benzyl-N-methyldithiocarbamate (BMDC) combines with metals to produce antimicrobial and anti-biofilm activity against methicillin-resistant Staphylococcus aureus (MRSA) and Staphylococcus epidermidis
Source: mSphere. 2025 Dec 11;11(1):e00691-25. doi: 10.1128/msphere.00691-25 (PMC12838442; doi:10.1128/msphere.00691-25)

Supplemental Figure 1. A) Structure of BMDC B) BMDC in complex with Copper (II) or Zinc (II).

Supplemental Figure 2. Growth curve of MRSA and survival assay. A) MRSA growing on RPMI under increasing copper concentrations. B) Same as A, but increasing zinc concentrations. C) Survival assay of MRSA. Bacteria were grown to a concentration of 10^8 CFU/mL in RPMI. Then 32 µM of BMDC was added with either 500 µM copper or 500 µM zinc, and samples were collected at predetermined times. Another experiment involved adding 500 µM of zinc to the culture, along with 32 µM BMDC. This culture was incubated for 4 hours, then 500 µM of copper was added. Bacterial samples were taken at predetermined times.

Supplemental Figure 3. ICP-OES metal values in MRSA pellets 60- or 120-minutes post-metal-compound exposure. A) MRSA was exposed to treatments for two hours, and levels of copper were determined in the pellet. B) Same as A, but zinc concentrations. C) Concentration of manganese at 120 minutes post-treatment. D) Concentration of magnesium at 120 minutes post-treatment. Statistical differences were measured by an unpaired t-test with Welch's correction (A-B) or one-way ANOVA with Dunnett's multiple comparison test against the untreated control (C-D) (ns, non-significant; *, P < 0.05; **, P <0.01; ***, P <0.001; ****, P <0.0001).

Supplemental Figure 4. Bacterial counts after peroxide and resazurin assays. A) Incubations in copper. B) Incubations in zinc. Bacteria were incubated for 1 hour with the indicated treatments and an untreated control. Bacterial counts were performed at

the end of the testing period. Statistical differences were measured using a one-way ANOVA with Dunnett's multiple comparison test against the untreated control (ns, non-significant; *, P < 0.05; **, P <0.01; ***, P <0.001; ****, P <0.0001).

Supplemental Figure 5. Biofilm quantification using safranin staining. Statistical differences were measured with an unpaired t-test with Welch's correction (ns, non-significant; *, P < 0.05; **, P <0.01; ***, P <0.001; ****, P <0.0001).

Supplemental Figure 6. BMDC sensitizes bacteria in biofilm to metal intoxication with a 6-hour delay in exposure. A) After establishing biofilm and adding the respective metal for 6 hours, followed by 32 µM BMDC addition and incubation for 24 hours, CFU were measured in the supernatant. B) Same as A, but quantifying CFU in the biofilm fraction. C) ICPOES data from biofilms showing copper accumulation in indicated treatments after adding BMDC at 6-hour post-exposure and incubating for 24 hours. Statistical differences were measured with a one-way ANOVA Barlett's test and Dunnett's multiple comparisons test (ns, non-significant; *, P < 0.05; **, P <0.01; ***, P <0.001; ****, P <0.0001).

Supplemental Figure 1.

A.

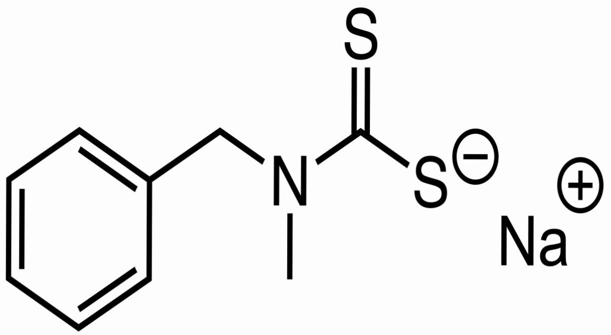

BMDC

B.

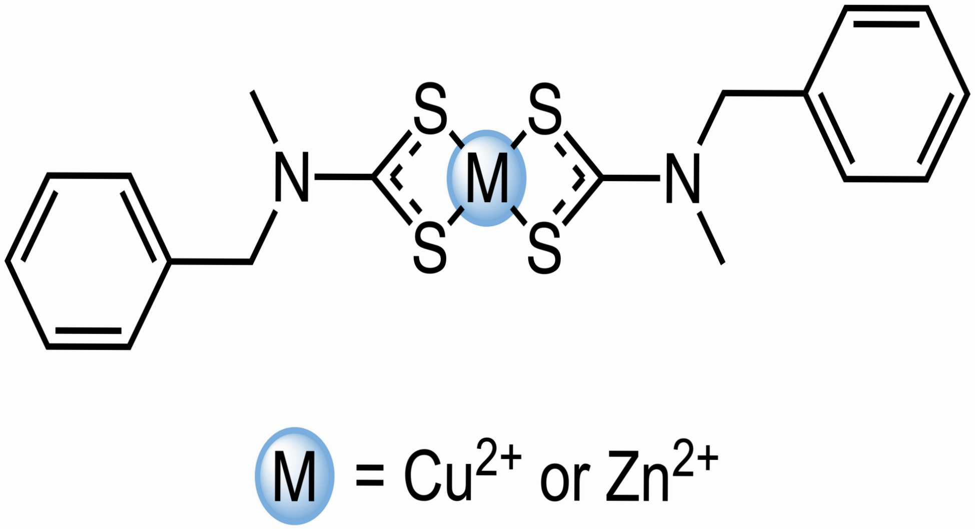

Supplemental Figure 2.

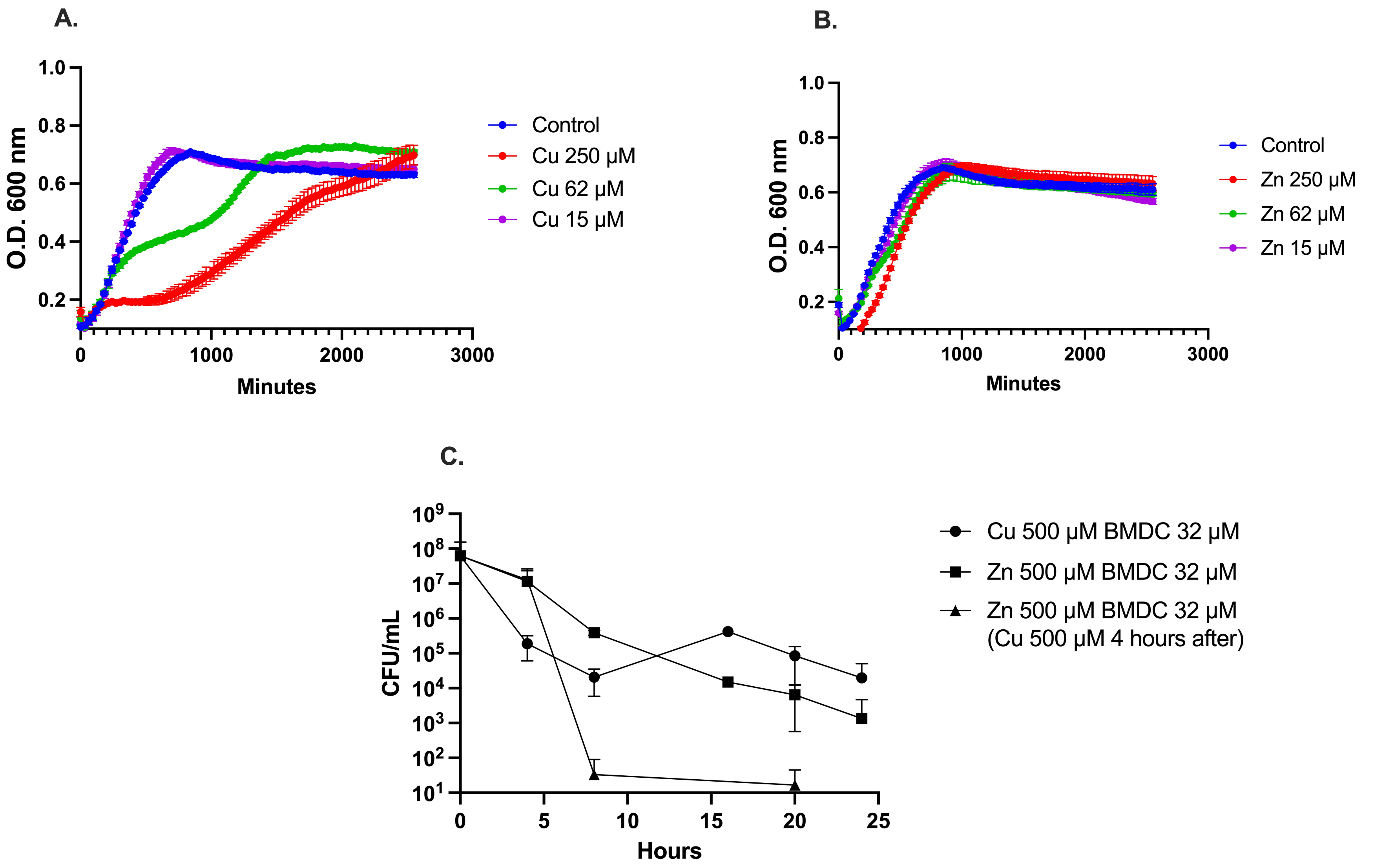

Supplemental Figure 3.

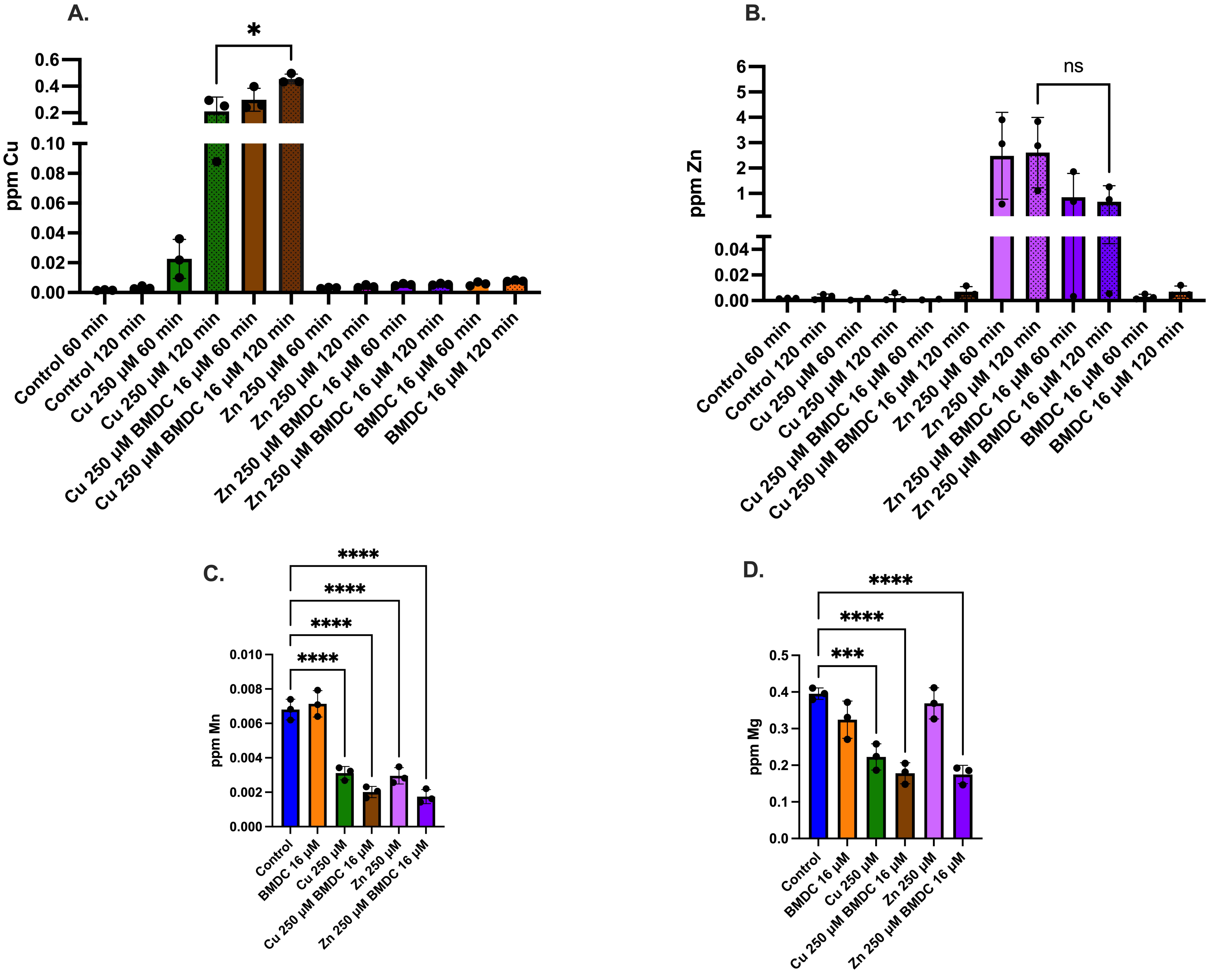

Supplemental Figure 4.

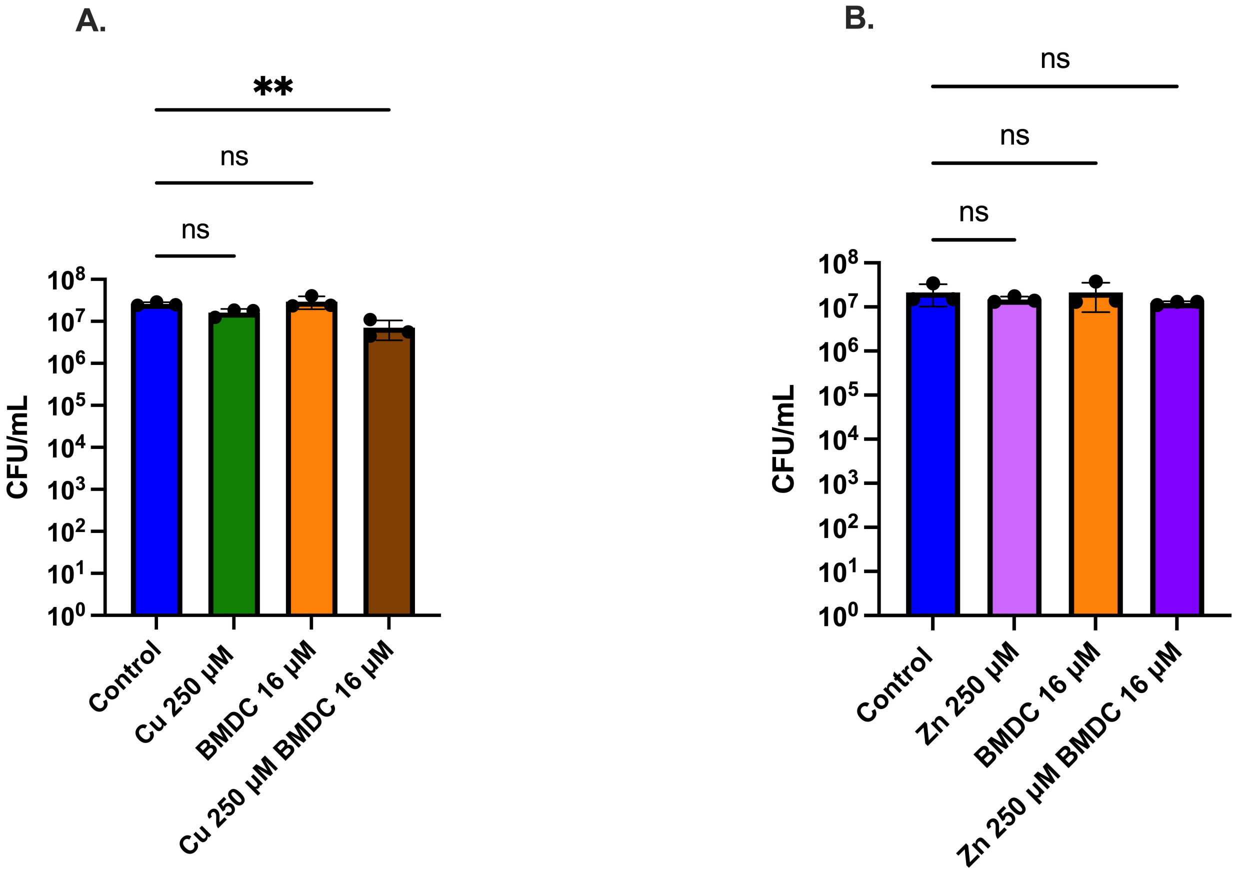

Supplemental Figure 5.

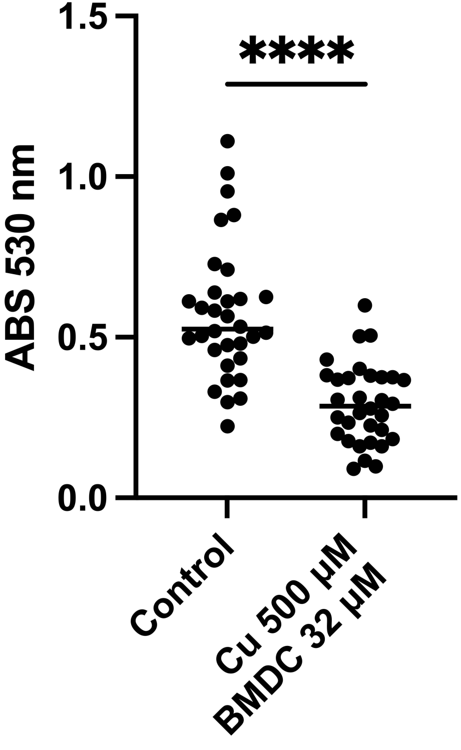

Supplemental Figure 6.

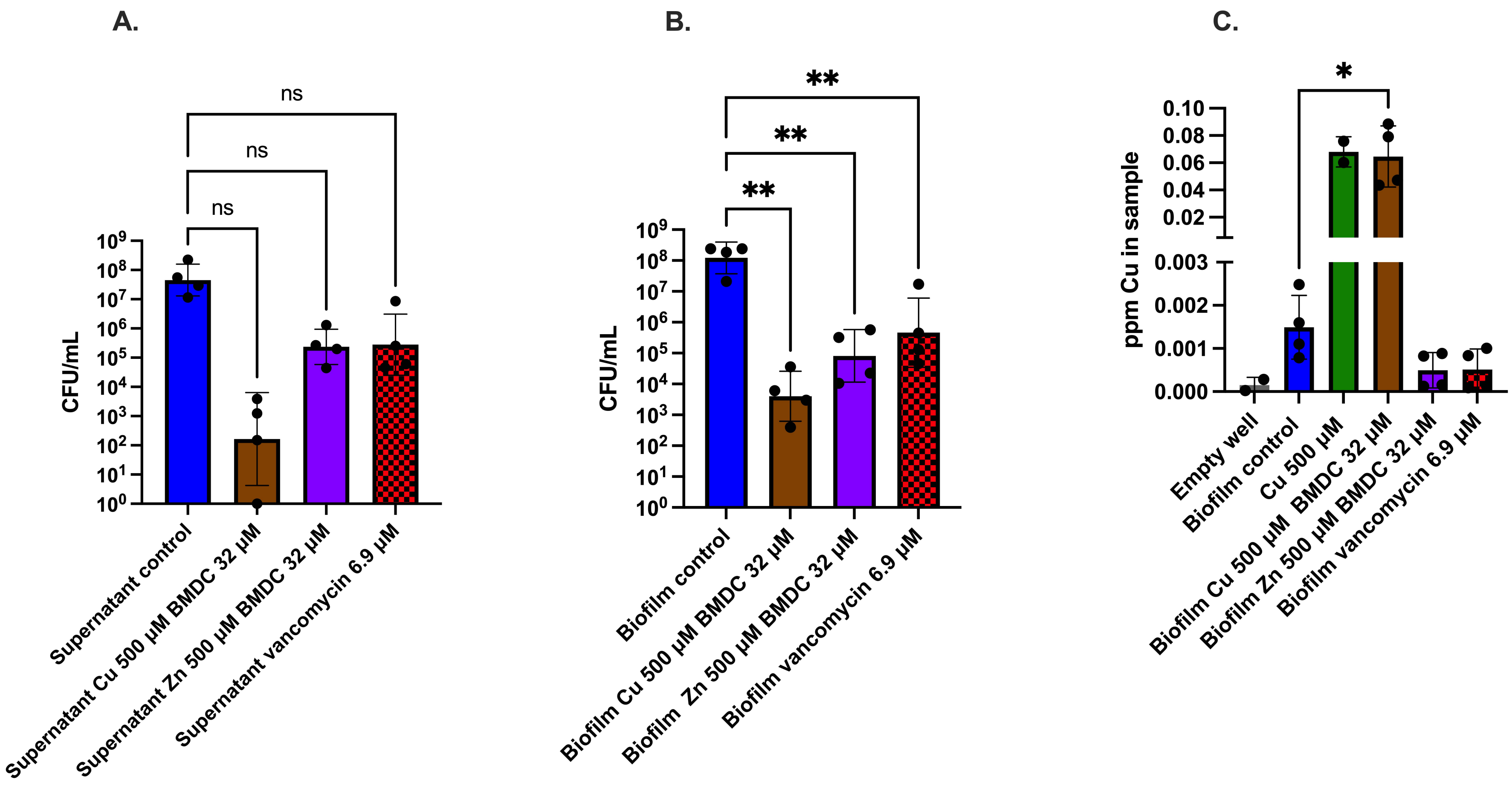

Supplement: Supplemental figures — Figures S1-S6. [file msphere.00691-25-s0001.pdf]
